# Cardiovascular Disease in Rheumatoid Arthritis: A Systematic Literature Review in Latin America

**DOI:** 10.1155/2012/371909

**Published:** 2012-10-31

**Authors:** Juan Camilo Sarmiento-Monroy, Jenny Amaya-Amaya, Juan Sebastián Espinosa-Serna, Catalina Herrera-Díaz, Juan-Manuel Anaya, Adriana Rojas-Villarraga

**Affiliations:** Center for Autoimmune Diseases Research (CREA), School of Medicine and Health Sciences, Universidad del Rosario, 111221 Bogotá, Colombia

## Abstract

*Background*. Cardiovascular disease (CVD) is the major predictor of poor prognosis in rheumatoid arthritis (RA) patients. There is an increasing interest to identify “nontraditional” risk factors for this condition. Latin Americans (LA) are considered as a minority subpopulation and ethnically different due to admixture characteristics. To date, there are no systematic reviews of the literature published in LA and the Caribbean about CVD in RA patients. *Methods*. The systematic literature review was done by two blinded reviewers who independently assessed studies for eligibility. The search was completed through PubMed, LILACS, SciELO, and Virtual Health Library scientific databases. *Results*. The search retrieved 10,083 potential studies. A total of 16 articles concerning cardiovascular risk factors and measurement of any cardiovascular outcome in LA were included. The prevalence of CVD in LA patients with RA was 35.3%. Non-traditional risk factors associated to CVD in this population were HLA-DRB1 shared epitope alleles, rheumatoid factor, markers of chronic inflammation, long duration of RA, steroids, familial autoimmunity, and thrombogenic factors. *Conclusions*. There is limited data about CVD and RA in LA. We propose to evaluate cardiovascular risk factors comprehensively in the Latin RA patient and to generate specific public health policies in order to diminish morbi-mortality rates.

## 1. Introduction

 RA is the most common inflammatory arthropathy worldwide with a prevalence of 0.5–1.0% in industrializedcountries [1]. The annual incidence is highly variable (12 to 1,200 per 100,000 population) and is dependent on a variety of factors, including sex, ethnicity, and age [2]. RA is a chronic, multiorganic, and complex disease with an autoimmune basis. The disease is three times more frequent in women than men [1]. RA can damage virtually any extraarticular tissue due to a systemic proinflammatory state. Cardiovascular disease (CVD) is considered an extraarticular manifestation (EAM) [3] and a major predictor of poor prognosis [2]. Several studies have documented a high prevalence of CVD in many autoimmune diseases (ADs) [2, 4–14]. Several traditional risk factors such as obesity, dyslipidemia, type 2 diabetes mellitus (T2DM), metabolic syndrome (MetS), hypertension, physical inactivity, advanced age, male gender, family history of CVD, hyperhomocysteinemia, and tobacco have been associated with CVD in RA patients [15–20]. In fact, seropositive RA may, like diabetes, act as an independent risk factor for CVD [21]. A proinflammatory state [7], insulin resistance [22], hyperhomocysteinemia [23], and oxidative stress [24] are common characteristics of both RA and atherogenesis. Nevertheless, excessive cardiovascular events observed in RA individuals are not fully explained by these traditional risk factors [7, 24]. Hence, there is an increasing interest in identifying “nontraditional” [4, 5] novel risk factors (i.e., genetic polymorphisms, autoantibodies, medication, duration of RA, high disease activity, development of EAM and many others) in order to explain the development of early endothelial dysfunction, increased intima-medial thickness (IMT), and finally, accelerated atherosclerosis [25]. The finding and understanding of these predisposing factors will allow us to better describe cardiovascular subphenotypes including hypertension, stroke, coronary artery disease (CAD), angina, myocardial infarction (MI), arrhythmias, ventricular diastolic dysfunction [26, 27], congestive heart failure (CHF), thrombosis, and peripheral arterial disease [16, 28].

Life expectancy of patients with RA is three to ten years less than that of the general population [29]. Although it is well established that cardiovascular mortality is higher in RA, the reasons for this remain elusive [30]. Currently, ischemic heart disease (IHD) secondary to atherosclerosis is the most prevalent cause of death associated with CVD in patients with RA [31]. CVD accounts for 30–50% of all deaths in RA patients [3]. Thus, RA added to CVD as the leading cause of death around the world [32, 33] requires us to take these diseases more seriously. Therefore, doctors need to be more committed to assessing, monitoring, and treating cardiovascular risk factors in the early stages as well as to promoting lifestyle changes in order to diminish morbi-mortality rates in RA individuals.

Hispanics are considered a minority group due to a mixed ethnicity (so called *mestizos*) that is mainly derived from a European and Amerindian inheritance [34]. Therefore, they represent a unique population. So far, some studies of RA have documented differences in health status, disease prevalence, treatment outcomes, and healthcare use among different ethnic groups [35, 36] which suggest that minority health disparities influence RA. Moreover, CVD is still one of the most important comorbidities in this subpopulation due to augmented mortality secondary to accelerated atherosclerosis, systemic inflammation, and MI or stroke [37–39].

RA is not uncommon in LA, the geographical area defined by Mexico, Central America, South America, and the islands of the Caribbean [1]. Overall,RA affects 0.5% of LA [40]. In Argentina, Spindler et al. [41] reported an overall prevalence ratio (per 1,000) of 1.97 (95% CI: 1.8–2) for both sexes, 0.6 (95% CI: 0.49–0.73) for men and 3.2 (95% CI: 2.9–3.5) for women. Peláez-Ballestas et al. [42] found a prevalence of 0.7–2.8% in Mexican patients. In an isolated African Colombian population, a prevalence of 0.01% was reported [43]. However, CVD has not been systematically assessed in LA and only a few studies have evaluated some of the traditional and nontraditional risk factors, cardiovascular subphenotypes, and mechanisms underlying the accelerated atherosclerosis that is characteristic of this population. Therefore, in this study, a systematic review of CVD in LA patients with RA was done.

## 2. Material and Methods

### 2.1. Search Strategy

A systematic literature review of articles on CVD and RA in LA was carried out in the following databases: PubMed, LILACS, SciELO, and Virtual Health Library (VHL). It included articles published between January 1947 and May 2012. Two reviewers did the search independently (SMJC and HDAC) while applying the same selection criteria described below. The search results were compared and disagreements were resolved by consensus. The Preferred Reporting Items for Systematic Reviews and Meta-Analyses (PRISMA) guidelines were followed in data extraction, analysis, and reporting [44].

The search was done in PubMed, using the following Medical Subject Headings (MeSH terms): “Arthritis, Rheumatoid,” “Latin America,” “Ethnic Groups,” “Minority Groups,” “Latin America/Epidemiology,” “Latin America/Ethnology,” “Brazil,” “Mexico,” “Colombia,” “Chile,” “Cuba,” “Panama,” “Venezuela,” “Bolivia,” “Peru,” “Argentina,” “Uruguay,” “Paraguay,” “Ecuador,” “Nicaragua,” “Surinam,” “French Guiana,” “Guatemala,” “Honduras,” “Belize,” “Costa Rica,” “El Salvador,” “Puerto Rico,” “Dominican Republic,” and “Haiti.” Each one of them was cross-referenced with the following MeSH terms: “Cardiovascular Diseases,” “Hypertension,” “Thrombosis,” “Stroke,” “Myocardial Infarction,” and “Coronary Artery Disease.” Each term was cross-referenced for the greatest number of results. No limits regarding language, period of publication, or publication type were used. In a quality control assessment of the first systematic search, it was evident that some publications were missed when only MeSH terms were used. Therefore, a second search was done by implementing key words. In the second search, also without limits, MeSH terms (“Hispanic Americans” and some of the previously described terms such as “Arthritis, Rheumatoid;” “Latin America” and “Minority Groups”) and key words (Rheumatoid Arthritis was matched with every country and Hispanics with RA) were included.

A similar strategy was followed for the other databases. Each MeSH term was translated into DeCS (Health Sciences Descriptors) in order to explore sources of information in Portuguese, Spanish, and English through SciELO, LILACS and VHL databases. The following terms were selected: “Artritis Reumatoide,” “América Latina,” “Salud de Minorias,” “Grupos Étnicos,” “Brasil,” and “Haití” (24 countries, as well as PubMed). Then each of the terms was cross-referenced with the following: “Enfermedades Cardiovasculares,” “Hipertension,” “Embolia y Trombosis,” “Accidente Cerebrovascular,” “Infarto del Miocardio,” and “Enfermedad Coronaria” for the first search. Each term was cross-referenced for the greatest number of results. Once again, no limits were used. For the second search in SciELO, some of the DeCS terms and keywords included were Artritis Reumatoid, América Latina, Salud de Minorias, Grupos Étnicos, “Enfermedades Cardiovasculares,” “Hipertension,” “Embolia y Trombosis,” “Accidente Cerebrovascular,” “Infarto del Miocardio,” and “Enfermedad Coronaria.” Both Spanish (Artritis Reumatoide) and English (Rheumatoid Arthritis) key words were matched with every country (Brazil to Haiti). “Artrite Reumatoide” was included as an additional term for Brazil in the search for articles published about CVD in this country. Likewise, in two remaining databases—LILACS and VHL (all sources)—both Spanish (Artritis Reumatoide) and English (Rheumatoid Arthritis) key words were matched with every country (Brazil to Haiti). As in SciELO, “Artrite Reumatoide” was included as an additional term for Brazil.

### 2.2. Study Selection, Data Extraction, and Quality Assessment

A study was included if (a) the abstract was available, (b) it contained original data, and (c) it used accepted classification criteria for RA and measured cardiovascular risk factors (traditional, nontraditional) and/or any of the cardiovascular subphenotypes. Articles were excluded from the analysis if they dealt with juvenile idiopathic arthritis or were done on animal models (i.e., murine models) instead of RA patients. Studies were also excluded if they were reviews or case reports, if they discussed topics not related to CVD, and/or were not done on an LA population. Those references from the articles that seemed to be relevant for the present paper were hand-searched and were included in the discussion. Abstracts and full text articles were reviewed to find eligible studies. Duplicate papers were excluded.

 Three blinded reviewers (SMJC, AAJC, and HDAC) organized selected articles on the basis of publication source, author, cardiovascular outcome, and traditional and nontraditional cardiovascular risk factors as well as subphenotypes evaluated. Moreover, a descriptive analysis from these data was completed. Articles were not included in the analysis when there was a lack of inclusion criteria, insufficient data, and statistical significance. A database with pertinent information from these studies which included authors, name of study, country, language, study design, number of patients, objective, cardiovascular outcome, method of hypothesis testing, results, limits/bias of the study, and reference was created. Disagreements between the reviewers were resolved by consensus. Each record was classified based on the quality score of the studies that was assigned by applying the levels established by the Oxford Centre for Evidence-based Medicine 2011 in order to evaluate the risk of bias [45].

## 3. Results

### 3.1. Systematic Literature Review

**Figure 1:**
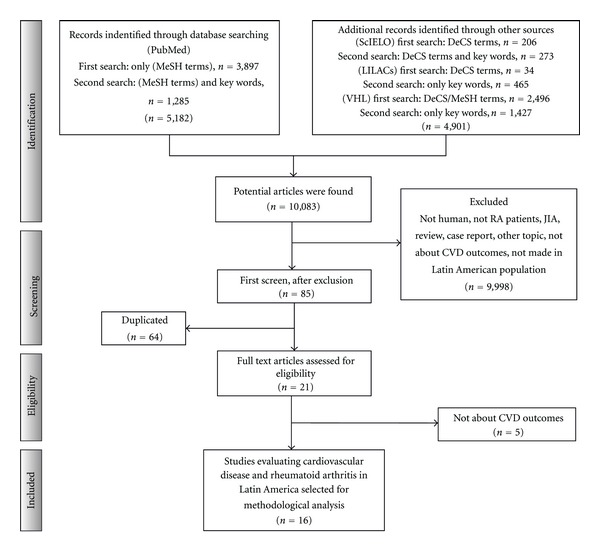
Flow chart of the systematic literature review; VHL: virtual health library; RA: rheumatoid arthritis; JIA: juvenile idiopathic arthritis; CVD: cardiovascular disease.

There were 3,897 articles identified in the first and 1,285 articles in the second search in PubMed (total of 5,182). Additional records identified through other sources included 206 articles from SciELO in the first search and 273 in the second one, 34 and 465 from LILAC, and 2,496 and 1,427 from VHL. Therefore, the database searches provided a total of 10,083 publications. Of these, 9,998 studies were discarded because they did not meet the eligibility criteria. After this exclusion, 85 articles were assessed and duplicates were identified (64 papers). A total of 21 full text articles were assessed for eligibility. Finally, only 16 articles [25, 30, 46–59] that had interpretable data and fulfilled the eligibility criteria were included. Of the selected articles, there were 5 from Mexico, 3 from Brazil and Colombia, 2 from Argentina, and 1 from Chile, Cuba and Puerto Rico, respectively. Seven were cross-sectional, 6 were case controls, 2 descriptive/retrospective, and only one corresponded to a cohort study. Half the studies had a sample size that was less than 100 patients. The flow chart for systematic literature review and articles included in the analysis are shown in Figure 1.

### 3.2. Risk Factors, Physiopathological Changes, and Subphenotypes of CVD

The prevalence of CVD in LA patients with RA was 35%. Several *traditional* cardiovascular risk factors such as dyslipidemia [25, 30, 48, 50, 51, 53, 55, 56, 58, 59], hyperhomocysteinemia [30, 48], smoking [25, 30, 48, 50], T2DM [25, 48, 50, 53, 56, 58], MetS [25, 50, 53], hypertension [30, 48–50, 52–56, 58], male gender [25, 46, 48, 49, 52–55], obesity [25, 49, 50, 52], physical inactivity [25, 50], and family history of CVD [25, 55] were reported. Several studies were associated with *nontraditional* risk factors, previously described in the literature, such as HLA-DRB1 shared epitope (SE) allele [25], rheumatoid factor (RF) [25, 30, 46, 49, 52, 55, 57, 58], anticyclic citrullinated peptide antibody (anti-CCP) [25, 55], and other autoantibodies [51]. These included anticardiolipins (aCL), anti-*β*2glycoprotein I (anti-*β*2GPI), antioxidated low-density lipoprotein (anti-oxLDL), and antiheat shock proteins 60/65 antibodies (anti-HSP 60/65) [52]. Other nontraditional factors include long duration of RA (>10 years) [25, 55], markers of chronic inflammation such as C-reactive protein (CRP) and erythrocyte sedimentation rate (ESR) [48, 49, 52, 55], high disease activity score-28 (DAS-28) [25, 49, 52, 57] and simplified disease activity index (SDAI) [52], presence of EAM [25, 46, 55, 57], medications like methotrexate (MTX) [25, 30, 49], and steroids [25, 30, 55–57, 59]. The last factors described were thrombogenic factors such as von Willebrand factor (vWF) [49] and fibrinogen [52], and novel risk factors like poliautoimmunity (defined as the presence of more than one autoimmune disease in a single patient) [25, 46, 55, 58], and familial autoimmunity [25] (diverse autoimmune diseases cooccurring within families). These factors and their respective prevalence or associations are depicted in Table 1.

**Figure 2:**
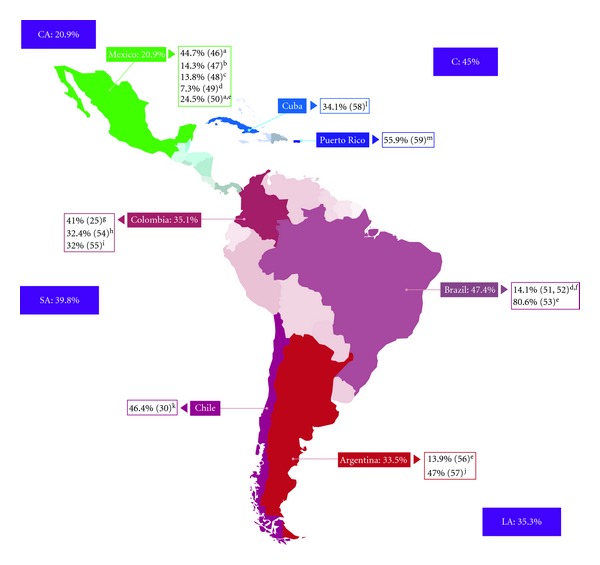
Cardiovascular disease in rheumatoid arthritis in Latin America and the Caribbean; LA: Latin America; CA: Central America; SA: South America; C: Caribbean. ^a^General cause of death was evaluated. CVD was the highest. ^b^Subclinical coronary artery disease. ^c^Hypertension and stroke. ^d^Not CVD subphenotype measured. Prevalence regarding presence of atherosclerosis plaque. ^e^Hypertension ^f^References [48, 49] report data from the same cohort of patients. Hence, the prevalence of CVD and risk factors is identical. ^g^Hypertension and atherosclerosis plaque. ^h^Hypertension and coronary artery disease. ^i^Hypertension and thrombosis. ^j^Ventricular diastolic dysfunction. ^k^Hypertension, stroke, and stable angina. ^l^Hypertension, stroke, coronary artery disease, peripheral vascular disease. ^m^Hypertension, myocardial infarction, angina pectoris, stroke, and peripheral vascular disease, and congestive heart failure.

Many groups described endothelial dysfunction, an increased IMT, and atherosclerosis plaque in RA patients [25, 49, 51, 52]. A broad spectrum of cardiovascular subphenotypes including stroke, CAD, MI, hypertension, thrombosis, peripheral arterial disease, and ventricular diastolic dysfunction were described in LA individuals with RA. Hypertension was the most common outcome in almost all studies with an overall prevalence of 28% (range 11.2–80.6%) [25, 48, 50, 53–56, 58, 59]. The average prevalence of CAD and stroke was 9% [47, 54, 58] and 2.5% [30, 46, 48, 58, 59], respectively.  Figure 2 shows the prevalences of CVD in LA and the Caribbean.

## 4. Discussion

To date, the literature evaluating CVD outcomes in LA individuals with RA is scarce. Only a few studies have assessed the classic and nontraditional risk factors in this subpopulation. 

### 4.1. Cardiovascular Disease as the Leading Cause of Mortality in LA

CVD is the leading cause of mortality worldwide. On the American continent, the prevalence and incidence of CVD is growing at an alarming rate. The World Health Organization (WHO) forecasts that the number of deaths in the region attributed to CVD will increase by more than 60% between 2000 and 2020 unless preventive measures are taken [60]. Thus, this chronic disease is one of the major causes of death around the world [33]. Thanks to the CARMELA initiative study, many traditional factors have been described in LA population such as hypertension, dyslipidemia, obesity, smoking, T2DM, and MetS [61].

Table 2, which was adapted from the Pan American Health Organization report [62], shows the mortality rates of CVD in the Americas as of 2007–2009 in terms of IHD and cerebrovascular disease. The data on this table is organized by country and region thus making it possible to contrast mortality rates from these two diseases in the United States of America (USA) and Canada with LA and the Caribbean. Generally, high rates of death were mostly observed in developed countries such as USA and Canada 136.3/100,000 people. Incidence of mortality in LA and the Caribbean due to IHD and cerebrovascular disease is 55.8/100,000 and 44.8/100,000 people, respectively. Individuals living in developed countries have more risk factors, for example, inappropriate life styles, that contribute to a higher rate of death from CVD. Thus, it is important to promote healthy habits among the general population and in patients with an early diagnosis of RA in order to prevent CVD. In specific LA countries, numbers show high rates of IHD in countries such as Cuba (140.1/100,000 people) and Puerto Rico (100.7/100,000 population). The importance of the numbers lies in the fact that they can be analyzed from the perspective of increased risk of CVD in RA in comparison to the general population. Therefore, it is important to discriminate mortality CVD rates by patients with chronic inflammatory diseases (i.e., RA).

LA has a growing population and it is a very dynamic region with an estimated population of 515 million. As mentioned before, the RA prevalence reported in LA is considered to be less than 0.5% [63, 64]. The heterogeneity across LA is expected due to the high degree of admixture between subpopulations. Hispanic/Latino populations are the result of a two-way admixture between Amerindian and European populations or of three-way admixture of Amerindian, European, and West African populations [65].

 Some studies have documented differences in the health status of, disease prevalence in, treatment outcome in, and healthcare use by different ethnic groups. Yazici et al. [35] compared patients from different ethnic groups with early RA using disease activity measures, identifying possible differences in patterns of clinical severity. They found that Hispanic patients with RA scored the worst in all self-report measures compared to Caucasians and African Americans with statistically significant differences in the Modified Health Assessment Questionnaire (MHAQ) functional score, psychological distress, and morning stiffness [35]. In a study of RA patients, Bruce et al. [36] demonstrated disparities between Caucasians and African Americans and Hispanics in disability, pain, and global health. Pain was worse in the latter two groups and global health was worse in Hispanics. The results of this exploratory study suggest that in a relatively similar cohort of patients with RA, minority health disparities exist [36]. Moreover, the prevalence of MI is high in Hispanics living in the USA, and coronary events are presented by people younger than in other minorities [48].

Nevertheless, only two studies in LA assessed mortality in RA patients. Orozco-Alcalá et al. [46] showed that there were no differences between RA patients and the general population concerning causes of death. Acosta et al. [58] demonstrated a mortality rate of 5.2% in a six-year followup. For both, the most frequent cause of death was CVD in 44.7% and 22.2% of the cases, respectively. In the other selected articles, a wide range of prevalence for CVD was reported (13.8–80.6%). The highest prevalence was indicated by Santiago-Casas et al. [59] in Puerto Rican patients (55.9%) when the demographic characteristics, clinical manifestations, comorbidities, pharmacological profile, and functional status of different age groups were determined. Nevertheless, the fact that elderly people (>60 years) have a higher probability of developing CVD whether or not they have RA had to be taken into account for calculating the prevalence of CVD in Puerto Rico. Cisternas et al. [30] evaluated cardiovascular risk factors in Chilean patients with RA and reported a prevalence of 46.4% for CVD. For Brazil [51, 53], Colombia [25, 54, 55], and Argentina [56, 57], a similar prevalence was indicated (47.4, 35.1 and 30.5%, resp.). In Mexico, five studies [46–50] reported an overall prevalence of 20.9% for CVD in RA patients.

### 4.2. Traditional Risk Factors, CVD, and RA

RA is a relatively frequent AD, which is chronic in nature, and these patients are doubly at risk of developing any CVD subphenotype with respect to the non-RA population [66, 67]. In fact, IHD secondary to atherosclerosis is the most prevalent cause of death associated with CVD in patients with RA [30]. The worldwide prevalence of hypertension in RA is between 49 and 77% [5]. It is considered the most common comorbiditiy in Hispanic patients with RA. The most frequent classic risk factor for CVD in this systematic literature review (with more than 2,000 RA patients included) was hypertension as well. Nevertheless, a lower prevalence (27.9%) than that reported previously in other countries was found. Many of these predisposing factors have been described in LA studies: hypertension [30, 36, 53–55, 58, 59, 61, 68, 69], T2DM [25, 48, 50, 53, 56, 58], dyslipidemia [25, 58, 59, 70], MetS [17, 25, 50, 53, 68, 69, 71], and hyperhomocysteinemia [22, 25, 48, 72]. For details, see Table 3.

### 4.3. Nontraditional Risk Factors, CVD, and RA

Since there is no classification system for nontraditional risk factors, we would like to propose one. Our recommendation is to divide them into genetic, AD associated, and others. The genetic group includes both HLA and non-HLA genes. HLA-DRB1 SE alleles are related to chronic inflammation, endothelial dysfunction, premature death, and CVD itself [25, 73–80]. The non-HLA genes include polymorphisms in the endothelin-1 and methylene tetrahydrofolate reductase genes. Endothelin-1 enhances CVD by endothelial dysfunction and hypertension [81]. Methylene tetrahydrofolate reductase has been related to atherosclerosis and the clinical response to some Disease-Modifying Antirheumatic Drugs (DMARDs) [82]. Others genes are TNFA rs1800629 and NFKB1-94ATTG ins/del polymorphisms. These are associated with predisposition to cardiovascular complications in patients with RA, as subclinical and accelerated atherosclerosis [83, 84]. However, other gene polymorphisms placed outside the HLA region and not strongly associated with susceptibility to RA and CVD. Rodríguez-Rodríguez et al. [85] showed a potential influence of the CCR5Δ32 deletion on the risk of CV disease among patients with RA. This may be due to a protective effect of this allelic variant against the development of vascular endothelial dysfunction.

The AD associated factors include a broad spectrum of autoantibodies as well as RA characteristics. The autoantibodies include RF [25, 49, 86], anti-CCP, aCL, anti-B2GPI, anti-HSP 60/65 [25, 30, 51, 55], and anti-oxLDL [30, 87, 88]. The RA characteristics are inflammatory basis [39, 89, 90], high disease activity [91], long duration [25], systemic involvement [56, 76, 92], treatment (systemic steroids) [93–95], and others, recently described, such as polyautoimmunity [25, 46, 55, 58] and familial autoimmunity [25].

Other issues, such as thrombogenic factors, which include vWF and fibrinogen levels, are related to CVD as well [49, 96, 97]. Several new cardiovascular risk factors in RA have received only modest attention and the different studies have shown contradictory results in LA patients. Each of these factors contribute to an impaired endothelial function, increased IMT, accelerated atherosclerosis, and finally, manifest CVD. For details, see Table 3. 

### 4.4. Discovering Novel Nontraditional Risk Factors

Despite of all the traditional risk factors that have been associated with CVD in RA patients, the literature on it with respect to LA and the Caribbean is still scarce. Even though it has been generally accepted that systemic activity is related to chronic inflammation and accelerated pathogenic processes leading to cardiovascular compromise, it is important to assess other novel factors in patients that may also contribute. Therefore, we believe further research is needed in order to establish other factors that are not currently taken into account. To date, there are no systematic reviews of literature involving LA patients as a minority group.

After the systematic search was done, 2,119 RA patients from different LA countries were included and evaluated for cardiovascular outcomes in studies ranging from 1993 to 2012 (see Supplementary Table  1 in Supplementary Material available online at doi:1155/2012/371909). Common limiting factors in the sixteen studies analyzed included a lack of prospective follow up of RA patients and a general limitation on sample sizes. Most of the studies were either cross-sectional or case-control which in terms of evidence place them at level 4 [45]. Moreover, 50% of the studies included in the analysis had sample sizes of more than 100 RA patients. The rest of them had limited numbers of patients included, which was another common limit or bias found in the retrieved studies. Furthermore, the lack of adequate statistical methods and hypothesis testing in some of the studies should be noted. This was the case for four of the studies, which were descriptive or did not calculate *P* values, adjusted odds ratio or confidence intervals.

There is insufficient literature regarding CVD in LA patients with RA. Although the number of patients assessed is not negligible, when the geographical area of LA, the diversity, and the admixture of the population are considered, there is a need to include true cohorts to ensure more decisive conclusions.

### 4.5. Assessing CVD in RA Patients

Heartdisease in patients with RA is a major concern. Rheumatologists often face the question of how to treat and prevent CVD. To appropriately do so, we need to answer three important questions. *(1) How do we estimate the risk of CVD in RA? *Unfortunately, neither the Framingham Risk Score nor Reynold's Risk Score were designed to estimate risk in RA patients. The European League Against Rheumatism published their recommendation on estimating cardiovascular risk in RA; however, this has not been validated yet. *(2) Which actions decrease CVD risk?* Eating a well-balanced diet, exercising on a regular basis, quitting smoking, and maintaining a healthy weight have a positive impact on cardiovascular health. Targets based on the individual risk profile of every patient also have to be set. Well-established risk factors such as blood pressure, LDL levels, and hemoglobin A1C need to be considered. Treatments that reduce these risk factors include angiotensin-converting enzyme inhibitors, statins, and, in some patients, metformin. *(3) What should be the target of all these efforts?* That question raises more questions. Inflammation in RA is a risk factor for CVD which can be treated effectively, but can targeting “inflammation” decrease CVD risk in RA? Should the target be remission, a low CRP level, or lack of swollen joints? Is targeting specific inflammatory pathways more effective for reducing cardiovascular risk than other therapies? There are many unanswered questions and a lot of controversy about how to best address cardiovascular risk in patients with RA. Therefore, a comprehensive multidisciplinary approach is the first step towards addressing this complex issue and to optimize patient outcomes [98].

## 5. Conclusions

RA and CVD share common pathophysiology mechanisms (i.e., systemic and chronic inflammation) with secondary accelerated atherosclerosis that can explain the high mortality rates and augmented risk of ischemic events in these patients. Therefore, early or subclinical atherosclerosis should be assessed in every patient through the measurement of IMT in carotid arteries and other inflammatory markers on a regular clinical basis.

LA patients are ethnically different from other populations and have a worse disease course due to their different genetic burden that could be the cause of a higher prevalence of EAM. Trying to extrapolate previous results from countries with patients from a different ethnic group to our subpopulation could be a mistake.

Although there is an evident association of traditional risk factors and cardiovascular compromise in RA patients, they do not completely explain the high rates of CVD in these patients. Thus, novel risk factors which are related to autoimmunity are now becoming a more important focus of attention. This is the reason why we propose to separate traditional and nontraditional risk factors and evaluate them comprehensively and in a multidisciplinary fashion.

There is a lack of literature about CVD in Hispanic patients as demonstrated by this systematic search. To make matters worse, literature evaluating nontraditional risk factors is scarce. This should be a challenge to the rheumatologist to do research in these fields in order to elucidate the underlying mechanisms involved for the benefit of the patient.

Unfortunately, LA patients receive lower quality diagnostic assessment and treatment choices than Caucasian patients due to difficulties in access to health services and delayed diagnosis. Cardiovascular compromise in RA patients is a therapeutic challenge and doctors need to be committed to assessing, monitoring, and treating cardiovascular risk factors in the early stages as well as generating effective public health policies in developing LA countries so that morbi-mortality rates can be decreased promptly.

## Supplementary Material

A database with pertinent information from these studies which included authors, name of study, country, language, study design, number of patients, objective, cardiovascular outcome, method of hypothesis testing, results, limits/bias of the study, and reference was created. Disagreements between the reviewers were resolved by consensus. Each record was classified based on the quality score of the studies that was assigned by applying the levels established by the Oxford Centre for Evidence-based Medicine 2011 in order to evaluate the risk of bias [78].

## Figures and Tables

**Table 1:** Traditional, nontraditional risk factors, physiopathological changes, and subphenotypes of cardiovascular disease and rheumatoid arthritis in Latin America.

Ref.	Country	Author		*N*	Cardiovascular outcome measured			
			Prevalence of CVD in RA (%)		Traditional risk factors of CVD *n*/*N* (%)	Nontraditional risk factors of CVD *n*/*N* (%)	Physiopathological changes in CVD *n*/*N* (%)	Subphenotype of CVD described *n*/*N* (%)
[46]	Mexico	Orozco-Alcalá et al.^a^	44.7	38	Male gender 12/38 (31.6)	Polyautoimmunity 6/38 (15.8); RF 32/38 (84.2); EAM 16/38 (42.1); GC 9/38 (23.7); RA duration over 10 years 17/38 (44.7)	N/A	Stroke 10/38 (26.3), MI 3/38 (7.9), CHF 3/38 (7.9), acute pulmonary embolism 1/38 (2.6)
[47]		Zavaleta et al.	14.3^b^	37 (14 with RA)	Dyslipidemia^c^: TGL 106.2 ± 55.1^†^; male gender 2/14 (14.3)	RA duration 8.2 ± 7.1^c^	N/A	Alterations in myocardial perfusion: Subclinical CAD 2/14 (14.3)^b^
[48]		Lopez-Olivo et al.	13.8	152 RA patients/153 controls	Obesity: BMI 25 (16–36)^‡^, dyslipidemia: hypercholesterolemia 5/152 (3.3), T2DM 8/152 (5.3), male gender 26/152 (17.1)^§^, hyperhomocysteinemia 31/152 (20.4)^§^ and smoking 56/152 (36.8)^§^	RF 149 (0–3,050)^‡^; CRP (mg/dL) 17 (3–126)^‡§^; RADAR 1.0 (0.1–2.7)^‡^, HAQ-Di 0.7 (0–2.7)^‡^; MTX 14/31 (45.2)^d^	N/A	Hypertension 17/152 (11.2), stroke 4/152 (2.6)
[49]		Daza, et al.^e,f^	7.3^g^	55 RA patients/22 controls	Obesity: BMI 27.51 ± 4.05/24.48 ± 3.73^§^	RF 506.5 (347.1–665.8)^‡^/13.3 (5.8–20.8)^§^; CRP (mg/dL) 1.48 (1.05–1.9)^‡^/0.6 (0.02–1.2)^§^, ESR 28.47 ± 14.48^†^/9.65 ± 3.56^§^; DAS 28 4.77 (4.5–5.1)^‡^; MTX 45/55 (81.8), SSZ 6/55 (10.9), GC 8/55 (14.5); vWF 145.6 ± 30.1^†^/121.8 ± 37.2^§^	IMT 0.67 ± 0.18^†^/0.58 ± 0.10 (mm)^§^, atherosclerosis plaque 4/55 (7.3)	N/A
[50]		Zonana-Nacach et al.	24.5^#^	192 (107 RA and 85 SLE patients)	Obesity 49/192 (25.5), dyslipidemia 49/192 (25.5), T2DM 14/192 (7.3), MetS (18.7), physical inactivity 75/192 (87), and smoking 28/192 (14.6)	RA duration 135 ± 112^†^ (months); Housewife 102/192 (53)	N/A	Hypertension 47/192 (24.5)

[51]	Brazil	Pereira et al.^i^	14.1^g^	71 RA patients/51 controls	Male gender^j^ 7/71 (9.9)	CRP^k^ (mg/L): 8.64 ± 8.27^†^/19.75 ± 25.08, ESR^k^: 24.1 ± 14.6^†^/34.07 ± 23.54; DAS 28: 4.24 ± 1.02^†^/4.64 ± 1.05; SDAI^k^: 35.54 ± 12.34/48.5 ± 30.28^§^; MTX^k^ (mg/w): 20 ± 5/19.09 ± 5.74, GC^k^ (mg/day): 7.25 ± 2.75/7.72 ± 3.59; fibrinogen^k^ 326.04 ± 113.56^†^/371.94 ± 121.08^§^	IMT^j^: 0.72 ± 0.17/0.67 ± 0.15 (mm). Carotid plaquesj (IMT > 1.5 mm) 10/71 (14.1)/1 (1.9)^§^	N/A
[52]		Pereira et al.^l^	14.1^g^	71 RA patients/53 controls	Dyslipidemia^k^: CT 243.3 ± 31.2/191.54 ± 36.21^§^, male gender^j^ (9.9/13.9)	Same data from [48] and autoantibodies: RF^k^: 195.10 ± 281.71^†^/308.13 ± 584.46, anti-CCP (79/1.9), aCL (IgG 5.6/3.8, IgM 7/3.8), anti-B2GPI (IgG 1.4/1.9, IgM 4.2/1.9), anti-HSP 60 (14.1/7.5), anti-HSP 65 (36.6/17), anti-LPL antibodies (2.8/1.9)	IMT^j^: 0.72 ± 0.17/0.67 ± 0.15 (mm). Carotid plaquesj (IMT > 1.5 mm) 10/71 (14.1)/1 (1.9)^§^	N/A
[53]		de Cunha et al.	80.6^#^	283 RA patients/226 controls	Obesity: BMI 26.6 ± 5.1^†^/26.8 ± 4.3, dyslipidemia: HDL 58.9 ± 16.4^†^/52.7 ± 12.1^§^, LDL 109.9 ± 33.2/122.8 ± 37.7^§^, T2DM 32/283 (11.3)-6/226 (2.7)^§^, MetS 111/283 (39.2)-44/226 (19.5)^§^, male gender 50 (17.7)/34 (15)	N/A	N/A	Hypertension^m^ 228 (80.6)/95 (42)

[54]	Colombia	Pineda et al.^n^	32.4	41	Male gender 12/41 (29)	N/A	N/A	Hypertension (24.2), CAD 9/41 (8.2)
[25]		Rojas-Villarraga et al.^o^	41^#^	140	Obesity 23/140 (16), dyslipidemia 49/140 (35), T2DM 6/140 (4), MetS 61/140 (44), physical inactivity 119/140 (85), male gender 16/(23), family history of CHD 22/140 (16), ever smoking 61/140 (44)^§^, and history of hormone replacement therapy 10/140 (7)	Poly-autoimmunity 31/140 (22); family history of autoimmunity 29/140 (21)^§^; HLA-DRB1 SE 60/136 (46)^§^; RF 85/134 (63)^§^, anti-CCP antibodies 73/94 (78); CRP 5.9 ± 15^†^, ESR 38.9 ± 25.1^†^; DAS 28 4.4 ± 1.4^†^, HAQ 1.7 ± 0.7^†^; EAM 60/140 (43); MTX 131/140 (94), GC 131/140 (94); RA duration 13.8 ± 8.5^§^	Early endothelial dysfunction 44/140 (31)^§^, increased IMT 75/140 (54)^§^, atherosclerosis plaque 10/140 (7)^§^	Hypertension 57/140 (41)
[55]		Ortega-Hernandez et al.^o^	32	538	Dyslipidemia 64/534 (10), male gender 80/538 (15)	Polyautoimmunity 48/538 (9); RF 246/385 (64)^§^, anti-CCP antibodies 146/183 (80); CRP 8.65 ± 20.21^†^, ESR^†^ 38.86 ± 25.93§; EAM 113/538 (21); GC 39/486 (8); RA duration 12.53 ± 8.08^§^	N/A	Hypertension 128/534 (24), thrombosis 43/534 (8)

[56]	Argentina	Larroudé et al.	13.9^#^	137	Dyslipidemia 89/137 (65), T2DM 2/137 (1.45), and male gender N/A 11/137 (8)	GC 71/137 (51.8)	N/A	Hypertension 19/137 (13.9)
[57]		Lascano et al.^p^	47	32 RA patients/32 controls	N/A	RF 27/32 (84); ESR 28 ± 15^†^; DAS 28 4.3 ± 1.4^†^; EAM 8/32 (25); GC 21/32 (66); RA duration 10.2 ± 8.4^†^	N/A	Ventricular diastolic dysfunction 15/32 (47)

[30]	Chile	Cisternas et al.^q^	46.4	54 RA patients/32 controls	Obesity: BMI 26 (18–39)^‡^, dyslipidemia 18/54 (33), male gender 7/54 (13), family history of CVD 9/54 (17), hyperhomocysteinemia 38/54 (70)^§^, and smoking 21/54 (39)	RF 50/54 (92), aCL^‡^ IgM 3 (0.53–23)/1.6 (0.21–10.6) IgG 4.3 (0.3–85)/2.5 (0–12.3); CRP 0.73 (0.04–5.96)^‡^/0.31 (0.05–2.88)^§^, ESR 27 (3–99)^‡^; MTX 41/54 (75), GC 42/54 (77); RA duration 9.5 (0.2–32)^‡^	N/A	Hypertension 21/54 (39), stroke 2/54 (3.7), stable angina 2/54 (3.7)

[58]	Cuba	Acosta et al.^r^	55.9	172	Dyslipidemia 10/172 (5.8), T2DM 16/172 (9.3), and male gender 29/172 (16.9)	Polyautoimmunity 2/172 (1.1); RF 52/85 (61.1)	N/A	Hypertension 46/172 (26.7), stroke 1/172 (0.5), CAD 8/172 (4.6), and peripheral vascular disease 4/172 (2.3)

[59]	Puerto Rico	Santiago-Casas et al.^s^	56.1	214	Dyslipidemia^§^ (9.1)-(52.7)-(58.4) T2DM^§^ (9.1)-(52.7)-(58.4) MetS (18.2)-(39.6)-(43.4) Smoking (4.5)-(11.0)-(7.9)	RF (52.4)-(52.9)-(57.1); ESR (81.0)-(92.2)-(91); Steroids^§^ (54.5)-(78.0)-(82.2); RA duration^‡^ (3.4 ± 2.9)-(9.5 ± 8.2)-(13.6 ± 10.7)	N/A	Hypertension (13.6)-(40.7)-(76.2), MI (0)-(2.2)-(9.1), angina pectoris (0)-(1.1)-(4.0), stroke (0)-(1.1)-(8.0), peripheral artery disease (0)-(1.1)-(5.0), and CHF (0)-(1.1)-(5.0)

CVD: cardiovascular disease; RA: rheumatoid arthritis; RF: rheumatoid factor; EAM: extraarticular manifestations; GC: glucocorticoids; N/A: not available; MI: myocardial infarction; CHF: congestive heart failure; TGL: triglycerides; CAD: coronary artery disease; BMI: body mass index; T2DM: type 2 diabetes mellitus; CRP: C-reactive protein; RADAR: rapid assessment of disease activity in rheumatology; HAQ-Di: health assessment questionnaire disability index; MTX: methotrexate; ESR: Erythrocyte Sedimentation Rate; DAS-28: Disease Activity Score-28; SSZ: sulfasalazine; vWF: von Willebrand Factor; IMT: intima-medial thickness; MetS: metabolic syndrome; SDAI: simplified disease activity index; TC: total cholesterol; anti-CCP: anti-cyclic citrullinated peptide antibodies; aCL: anticardiolipins antibodies; anti-B2GPI: anti-*β*2glycoprotein I antibodies; anti-HSP 60/65: anti-heat shock proteins 60/65 antibodies; anti-LPL: antiLipoprotein lipase antibodies; HDL: high-density lipoprotein cholesterol; LDL: low-density lipoprotein cholesterol.

^a^Only descriptive study, which evaluated causes of mortality in adult patients with RA.

^b^By echocardiogram and gammagraphy.

^c^Data from patients with RA 14/37 (37.8).

^d^Data from patients with hyperhomocysteinemia (>15 *μ*mol/L).

^e^Exclusion criteria: patient with traditional cardiovascular risk factors.

^f^Only female were included, each with at least 5 years of duration of the disease and between 35 and 54 years of age.

^g^Not CVD subphenotype measured. Prevalence regarding presence of atherosclerosis plaque.

^h^Only female were included.

^i^Exclusion criteria: smoking, diabetes and hypertension pregnancy, renal failure, chronic hepatopathy, nephrotic syndrome, hypothyroidism and use of statins/fibrates.

^j^RA patients versus controls.

^l^Exclusion criteria: smoking, diabetes, and hypertension.

^m^High blood pressure was defined above 130/85 mmHg.

^n^The objective was to analyze causes and direct costs of hospitalization of Colombian patients with RA.

^o^Sample population was originally from Northwestern Colombia. They are considered ethnically different.

^p^Exclusion criteria: any symptoms of heart disease or risk factors for CVD.

^q^Subjects over 60 years were excluded.

^r^Only cohort, 6 years followup. Low mortality rate 9/32 (5.2%).

^s^Three age group (<40 y)-(40–59 y)-(>60 y). Elder people (>60 y) have more probability to develop CVD independent of RA.

^†^Mean ± standard deviation.

^‡^Median (interquartile range).

^#^Prevalence of CVD regarding the only subphenotype described.

^§^ *P* values < 0.05 were considered significant.

**Table 2:** Cardiovascular disease mortality in the Americas*.

Region	Annual deaths average	Mortality rate from IHD^a,b^	Mortality rate from cerebrovascular disease^a^
	(thousands)^a^	Total	Total
*Americas *	6,447.2	87.4	45.1
*North America *	2,885	136	45
Canada	262.8	109	41.4
United States of America	2,621.7	139	45.4
*Latin America and the Caribbean *	3,562.2	55.8	44.8
*Latin America *	3,510.8	56	44.9
Mexico	549.4	54.1	27.5
Central American Isthmus	226.1	41.9	24.4
Belize	1.2	30.9	25.7
Costa Rica	20.4	48.4	21.3
El Salvador	41	56	22.4
Guatemala	80.5	25.5	16.4
Honduras	37.5	N/A	N/A
Nicaragua	27.5	54.2	32.8
Panama	18.1	57.2	51.5
*Latin Caribbean *	270.5	N/A	N/A
Cuba	83.9	140	80.6
Dominican Republic	60.1	N/A	N/A
French Guiana	0.9	N/A	N/A
Haiti	90	N/A	N/A
Puerto Rico	29.1	101	40.1
*Andean Area *	722.5	58.7	35.7
Bolivia	72.9	N/A	N/A
Colombia	260.6	74.1	38.7
Ecuador	74.5	25.6	34.1
Peru	161.4	27.8	26.6
Venezuela	153.1	81.4	41
Brazil	1.261.1	60.4	62.2
Southern Cone	481.3	49.1	51.2
Argentina	315.6	46.8	48.2
Chile	98.2	47.1	46.8
Paraguay	36.1	50.3	55.5
Uruguay	31.3	85.4	103
*Non-Latin Caribbean *	51.3	63.4	63.8
Guyana	4.4	80.9	70.3
Suriname	3.8	47	72

^∗^Adapted from [62]. The values were obtained from “Corrected Mortality” data. These values were computed by applying a correction algorithm for mortality underregistration and a redistribution algorithm for deaths from ill-defined causes. The methodology used is presented in Health Statistics from the Americas. 2006 edition (http://www.paho.org/HSA2006).

^a^Values are expressed in incidence rates/100.000 population (2007–2009).

^b^IHD: ischemic heart disease.

N/A: not available.

**Table 3:** Traditional and non-traditional risk factors associated with cardiovascular disease and rheumatoid arthritis in Latin America.

Risk factor associated with CVD		Comments	Reference(s)
		*Traditional *	

	Hypertension	Increases the risk to suffer IHD or stroke with an important impact on mortality in patients with RA	[16]

	T2DM	Patients with RA have a similar risk of developing CVD when compared to the same risk in patients with T2DM. Unfortunately, when there is a coexistence of both diseases, this risk is increased by three times	[69]

	Dyslipidemia	Altered lipid profiles in RA patients are related with higher probability of IHD by accelerating atherosclerosis	[25, 70]

		Is characterized for an alteration in production/secretion of proinflammatory adipokines and leads to increased activity of RA and accelerating atherosclerosis	[68, 71]
	MetS	Studies about the prevalence of MetS in LA patients have not achieved definitive conclusions, although its presence has been directly associated with a worse prognosis	[53]
		In RA patients, was related with pain and functional status, suggesting disease activity. Therefore, a better control of disease activity may reduce CVD risk	[50]

	Hyperhomocysteinemia	Homocysteine is considered as biomarker for atherosclerosis and a risk factor related with CAD and stroke	[22, 72]
		There is still controversy about whether hyperhomocysteinemia is a causative agent of cardiovascular damage or only an epiphenomenon of inflammation	[48]
		A high prevalence of this biomarker in Mexican patients with RA had a statistical association with male gender and higher radiological damage	[48]
		High homocysteine concentration can be an important risk marker for CVD in Chilean patients with RA, as it was significantly associated	[30]

		*Nontraditional *	

		Related with chronic inflammation, endothelial dysfunction, and premature death for CVD	[73–75]
*Genetic *	HLA-DRB1 SE alleles	Associated with severe RA and with more EAM, high activity, and systemic inflammation	[74–77, 79]
		Being a carrier of a single copy of HLA-DRB1 SE were significantly associated with an increased risk of atherosclerotic plaque in RA Colombian patients	[25]

	Polyautoimmunity	Some articles included patients with poliautoimmunity, but no correlation with CVD subphenotypes was described	[25, 46, 55, 58]
	Familial autoimmunity	Was associated with presence of atherosclerotic plaque in RA Colombian patients.	[25]
		High titers have been established to be a predictor of CVD due to immune complex formation and tissue injury. It has been shown that such immune complexes from RF can be deposited in the endothelium and through inflammatory reactions generate endotelial disfunction and atherosclerotic process	[86]
	RF positivity	RF seropositivity was significantly associated with an increased risk of endothelial dysfunction in RA Colombian patients	[25]
		A statistical association between increased IMT, atherosclerosis plaque, and presence of RF was described in Mexican population with RA	[49]
	anti-oxLDL	Promote instability and rupture of the atheromatous plaque within the coronary arteries	[24, 88]
		Only one LA study evaluated this antibodies, but no correlation with CVD was found	[30]
	Other autoantibodies	The presence of plaques was higher in Brazilian patients with RA, but no correlation between IMT or plaques and autoantibodies were found	[51]
*AD associated *		Other autoantibodies were assessed in LA population, such as aCL, anti-*β*2GPI, anti-HSP 60/6, and anti-CCP antibodies with no association regarding CVD outcomes	[25, 30, 51, 55]
	Inflammatory markers	The association of inflammatory pathways with CVD is complex and is composed of several intermediate factors, including dyslipidemia, homocysteinemia, insulin resistance, and endothelial dysfunction	[89]
		May accelerate atherogenic processes, either by the accentuation of known pathways of plaque formation or by the onset of additional immune pathways	[90]
	Disease activity	The lipid profile in RA depends on disease activity. Higher disease activity leads to depressed levels of total cholesterol. However, HDL cholesterol levels are even more depressed, resulting in a more unfavourable atherogenic index	[90]
	Long duration of RA (>10 years)	Implies more time for chronic inflammatory process to generate sequelae such as atherosclerosis and endothelial dysfunction	[39]
		Were significantly associated with an increased risk of atherosclerotic plaque in RA Colombian patients	[25]
	EAM	Is an indirect indicator of disease severity and systemic compromise. Patients are considered to have three times higher risk to develop CVD	[55, 76]
	GC	Could enhance cardiovascular risk owing to their potentially deleterious effects on lipids, glucose tolerance, insulin production and resistance, blood pressure, and obesity. On the other hand, it may actually decrease the risk of atherosclerosis and CVD by suppressing inflammation, which paradoxically may improve glucose intolerance and dyslipidaemia	[93]

*Others *	Thrombogenic factors	vWF has been recognized to induce a procoagulant state Represent a biomarker of endothelial dysfunction	[96, 97]
		The measurements of the IMT together with the vWF serum levels could give valuable information about the artery status and the atherosclerosis process in early stages in Mexican patients with RA without cardiovascular risk factors	[49]

CVD: cardiovascular disease; IHD: ischemic heart disease; RA: rheumatoid arthritis; T2DM: type 2 diabetes mellitus; LA: Latin America; MetS: metabolic syndrome; SE: shared epitope; RF: rheumatoid factor; IMT: intima-medial thickness; anti-oxLDL: anti-oxidized low-density lipoprotein antibodies; aCL: anticardiolipins antibodies; anti-B2GPI: anti-*β*2glycoprotein I antibodies; anti-HSP 60/65: antiheat shock proteins 60/65 antibodies; anti CCP: anti-cyclic citrullinated peptide antibodies; HDL: high-density lipoprotein cholesterol; EAM: extra-articular manifestations; GC: glucocorticoids; vWF: von Willebrand factor.

## Abbreviations

CVD: Cardiovascular disease
RA: Rheumatoid arthritis
LA: Latin America
EAM: Extraarticularmanifestations
T2DM: Type 2 diabetes mellitus
MetS: Metabolic syndrome
AD: Autoimmune disease
IMT: Intima-medial thickness
CAD: Coronary artery disease
MI: Myocardial infarction
CHF: Congestive heart disease
IHD: Ischemic heart disease
VHL: Virtual health library
SE: Shared epitope
RF: Rheumatoid factor
Anti CCP: Anti-cyclic citrullinatedpeptide antibodies
aCL: Anticardiolipin antibodies
Anti-B2GPI: Anti-*β*2glycoprotein I antibodies
Anti-oxLDL: Antioxidized low-density lipoprotein antibodies
Anti-HSP 60/65: Antiheat shock proteins 60/65 antibodies
CRP: C-Reactive Protein
ESR: Erythrocyte sedimentation rate
DAS-28: Disease activity score-28
SDAI: Simplified disease activity index
MTX: Methotrexate
vWF: von Willebrand factor
WHO: World Health Organization
MHAQ: Modified Health Assessment Questionnaire
DMARDs: Disease nodifying anti rheumatic drugs.
